# Rapid Detection of *bla*_KPC_, *bla*_NDM_, *bla*_OXA-48-like_ and *bla*_IMP_ Carbapenemases in *Enterobacterales* Using Recombinase Polymerase Amplification Combined With Lateral Flow Strip

**DOI:** 10.3389/fcimb.2021.772966

**Published:** 2021-12-02

**Authors:** Fang Wang, Lei Wang, Huimin Chen, Na Li, Yan Wang, Yan Li, Wei Liang

**Affiliations:** ^1^ Department of Central Laboratory, The Second People’s Hospital of Lianyungang City (Cancer Hospital of Lianyungang), Lianyungang, China; ^2^ School of Biotechnology, Jiangsu University of Science and Technology, Zhenjiang, China

**Keywords:** Carbapenemase, *Enterobacterales*, recombinase polymerase amplification, rapid detection, false positive

## Abstract

The emergence of carbapenemase-producing *Enterobacterales* (CPE) infections is a major global public health threat. Rapid and accurate detection of pathogenic bacteria is essential to optimize treatment and timely avoid further transmission of these bacteria. Here, we aimed to develop a rapid on site visualization detection method for CPE using improved recombinase polymerase amplification (RPA) combined with lateral flow strip (LFS) method, based on four most popular carbapenemase genes: *bla*_KPC_, *bla*_NDM_, *bla*_OXA-48-like_, and *bla*_IMP_. All available allelic variants of the above carbapenemases were downloaded from the β-lactamase database, and the conserved regions were used as targets for RPA assay. Five primer sets were designed targeting to each carbapenemase gene and the RPA amplification products were analyzed by agarose gel electrophoresis. FITC-labeled specific probes were selected, combined with the best performance primer set (Biotin-labeled on the reverse primer), and detected by RPA-LFS. Mismatches were made to exclude the false positive signals interference. This assay was evaluated in 207 clinically validated carbapenem-resistant *Enterobacterales* (CRE) isolates and made a comparison with conventional PCR. Results showed that the established RPA-LFS assay for CPE could be realized within 30 min at a constant temperature of 37°C and visually detected amplification products without the need for special equipment. This assay could specifically differentiate the four classes of carbapenemases without cross-reactivity and shared a minimum detection limit of 100 fg/reaction (for *bla*_KPC_, *bla*_NDM_, and *bla*_OXA-48-like_) or 1000 fg/reaction (for *bla*_IMP_), which is ten times more sensitive than PCR. Furthermore, the detection of 207 pre-validated clinically CRE strains using the RPA-LFS method resulted in 134 *bla*_KPC_, 69 *bla*_NDM_, 3 *bla*_OXA-48-like_, and 1 *bla*_IMP_. The results of the RPA-LFS assay were in consistent with PCR, indicating that this method shared high sensitivity and specificity. Therefore, the RPA-LFS method for CPE may be a simple, specific, and sensitive method for the rapid diagnosis of carbapenemase *Enterobacterales*.

## Introduction

*Enterobacterales* are conditionally pathogenic bacteria that cause serious hospital-acquired infections (Feil, 2017). The spread of carbapenemase-producing *Enterobacterales* (CPE) has become a major global public health threat. Carbapenems have traditionally been used to treat infections caused by broad-spectrum beta-lactamase-producing *Escherichia coli* and *Klebsiella pneumoniae* and are still considered antibiotics to be used as a last resort (Nordmann et al., 2012; van Duin and Doi, 2017; Rochford et al., 2018; Lin et al., 2020). Carbapenemase-producing enzymes in these bacteria, which are capable of hydrolyzing all carbapenems, cephalosporins, and beta-lactams are the main cause of resistance to carbapenem antibiotics (Khan et al., 2017; Segagni Lusignani et al., 2020). Most carbapenemase genes are located on the metastable genetic elements, such as plasmids and integrons; thus, carbapenem resistance is easily transferred horizontally leading to rapid spread wordwide (Conlan et al., 2014; Bengtsson-Palme et al., 2018; Paveenkittiporn et al., 2021). Among these, *Klebsiella pneumoniae* carbapenemase (KPC), New Delhi metallo-β-lactamase (NDM), oxacillinase (OXA-48-like), and imipenemase (IMP) are the most prevalent carbapenemases in CPE (Han et al., 2020).

Rapid and accurate detection of carbapenemase genes is extremely important for preventing and monitoring infections and avoiding large-scale carbapenem-resistant bacterial infections outbreak. Currently, in clinical microbiology laboratories, the detection of carbapenemase-producing bacteria is primarily performed using phenotypic methods, such as Carba-NP test, combined disk test, and carbapenem inactivation method (Aguirre-Quiñonero and Martínez-Martínez, 2017; Nordmann et al., 2020; Tsai et al., 2020). These methods generally have disadvantages of a prolonged testing time, being complex to perform, and being prone to false negatives, whereby some clinical isolates only show low levels of resistance (Hansen, 2021). Rapid assays have been developed based on carbapenemases, including immunochromatographic NG-Test Carba5, RESIST-5 O.O.K.N.V., IMP K-SeT, MALDI-TOF, polymerase chain reaction (PCR), and quantitative PCR (Glupczynski et al., 2016; Foudraine et al., 2019; Vergara et al., 2020; Kanahashi et al., 2021). These methods reduce the detection time to a few minutes; however, they rely on sophisticated instrumentation and trained personnel, which limit widespread application, especially in source-limited area.

Recombinase polymerase amplification (RPA) was first proposed by Piepenburg et al. (Piepenburg et al., 2006) and uses recombinase activity to open the double strand of a DNA molecule and amplifies the DNA target using strand-displacing enzyme activity. Amplification can be completed within approximately 30–40 min at a temperature range of 37°C–42°C. The use of lateral flow strips (LFS) as endpoint visual readouts of the amplified DNA targets makes the method less device-dependent. The colored signal can be observed semi-quantitatively by the naked eye on the LFS using gold nanoparticles (AuNPs) that interact with the labeled isothermal amplification product (Wang et al., 2019; Xie et al., 2021). RPA-LFS has been successfully used for the molecular diagnosis of diseases caused by pathogenic bacteria, such as methicillin-resistant *Staphylococcus aureus*, *Mycobacterium tuberculosis* and *neo-cryptococcosis* (Yang et al., 2013; Law et al., 2018; Ma et al., 2019).

In this study, the false-positive signals from primer dimers were thoroughly eliminated by the introduction of specific probes and base substitutions with specific guidance in the primer and probe sequences. As a result, a rapid and accurate RPA-LFS method was established for the detection of four (*bla*_KPC_, *bla*_NDM_, *bla*_OXA-48-like_, and *bla*_IMP_) important carbapenemase genes in clinical CPE of CRE strains. The assay could be completed within 30 min under 37°C isothermal condition. The rapid identification of carbapenemase genes from 207 pre-validated clinical CRE isolates demonstrated the high specificity and sensitivity of the method. Thus, a simple, specific, and sensitive assay was established to provide a technical reference for the rapid detection of clinical carbapenemases.

## Materials and Methods

### Ethics Statement

This study was approved by the Medical Ethics Committee of the Second People’s Hospital of Lianyungang City (Permit Number: 2020005). The clinical strains were collected from 2020 to 2021 and isolated from sputum, urine, drainage fluid, or secretion samples. All the isolate samples were obtained written consent, on an institutionally approved document, from every patient.

### Source of the Strain

Four PCR-amplified and sequenced ‘standard strains,’ including *bla*_KPC_, *bla*_NDM_, *bla*_OXA-48-like_, and *bla*_IMP_ from *Klebsiella pneumoniae* were used to establish the RPA-LFS assay for carbapenemases. Seven other common pathogenic bacteria, including *Klebsiella pneumoniae*, *Escherichia coli*, *Pseudomonas aeruginosa*, *Acinetobacter baumannii*, *Streptococcus pneumoniae*, *Staphylococcus aureus*, and *Enterococcus faecalis* were used to validate the specificity of the RPA-LFS method. A total of 207 clinical strains with carbapenem resistance validated by the paper diffusion method were collected from 2020 to 2021 and verified that no duplicate isolates were from the same patient. These clinical isolates, including *K. pneumoniae*, *Escherichia coli*, *Enterobacter cloacae*, *Citrobacter freundii*, and *Serratia marcescens*, were used to validate the practical application of the RPA-LFS technology for the rapid detection of carbapenemases in *Enterobacterales* (**Table 1**). All strains were collected from the microbiology laboratory of the Second People’s Hospital of Lianyungang City and identified by MALDI-TOF mass spectrometer.

**Table 1:** Information of bacteria strains used in this study.

Species	Strain amount	Source	Sample type	Carbapenase gene
*K. pneumoniae*	1	Sputum isolated strain	Reference Strain	*bla*_KPC_
*K. pneumoniae*	1	Sputum isolated strain	Reference Strain	*bla*_NDM_
*K. pneumoniae*	1	Sputum isolated strain	Reference Strain	*bla*_IMP_
*K. pneumoniae*	1	Sputum isolated strain	Reference Strain	*bla*_OXA-48-like_
*K. pneumoniae*	1	Sputum isolated strain	Reference Strain	/
*E. coli*	1	Sputum isolated strain	Reference Strain	/
*P. aeruginosa*	1	Sputum isolated strain	Reference Strain	/
*A. baumannii*	1	Sputum isolated strain	Reference Strain	/
*S. pneumoniae*	1	Sputum isolated strain	Reference Strain	/
*S. aureus*	1	Sputum isolated strain	Reference Strain	/
*E. faecalis*	1	Sputum isolated strain	Reference Strain	/
*K. pneumoniae*	159	Sputum, urine, drainage fluid	Validations Strain	131 *bla*_KPC_, 24 *bla*_NDM_, 3 *bla*_OXA-48-like_, and 1 *bla*_IMP_
*E. coli*	33	Urine, Sputum, drainage fluid	Validations Strain	33 *bla*_NDM_
*E. cloacae*	8	Sputum, urine, secretion	Validations Strain	8 *bla*_NDM_
*C. freundii*	5	Urine, drainage fluid	Validations Strain	1 *bla*_KPC_, 4 *bla*_NDM_,
*S. marcescens*	2	Sputum	Validations Strain	2 *bla*_KPC_

### Genomic DNA Extraction

For reactions using purified genomic DNA as a template, genomic DNA was extracted using the Bacterial Genomic DNA Extraction Kit (Tiangen Biochemical Technology Co., Ltd., Beijing, China) and stored at −20°C for backup. If bacterial cultures were used as templates, bacterial DNA was extracted using the heated boiling method. The individual colonies were suspended in 50 μL Tris-EDTA buffers, boiled for 10 min, and centrifuged at 12000 × g for 10 min, after which the supernatant was used as the DNA template.

### Design of Primers for RPA Reactions

For the primer design method, sequences of all the isoforms of *bla*_KPC_, *bla*_NDM_, *bla*_OXA-48-like_ and *bla*_IMP_ genes were downloaded from GeneBank. Five pairs of primers for each carbapenemase gene detection were designed separately using Primer Premier 5.0 to capture all the variants based on the regions conserved among the isoforms. The primer design parameters were: size setting of 30–35, product size of 100–500 bp, GC content of 20%–80%, and Tm value setting of 50–100. Default settings were used for all other parameters. Five pairs of primers were selected for testing according to their scores from the highest to the lowest.

### RPA Procedure

For the RPA experiments, we used the TwistAmp Liquid DNA Amplification Kit (TwistDx Inc., Maidenhead, United Kingdom). A total of 50 μL of the reaction system was added to the tubes in the following order: 25 μL 2 × reaction buffer, 5 μL 10 × base mix, 2.5 μL 20 × core mix, 2.1 μL upstream primer (10 μM) and 2.1 μL downstream primer (10 µM; General Biosystems Co., Ltd., Anhui, China), 9.8 μL ddH_2_O, and 1 μL template. To ensure that all reaction systems reacted simultaneously, 2.5 μL of 280 mM magnesium acetate was added to the PCR tube caps, and the template and 280 mM magnesium acetate were added to the reaction system simultaneously using transient centrifugation. The reaction system was vortex-centrifuged and immediately incubated in a heater at 37°C for 30 min. No genomic DNA template reaction system was used as negative control. Each sample has two tubes of reaction, one for the sample itself and the other for the control. Amplification of primers was detected using 1.5% agarose gel electrophoresis.

### RPA-LFS Probe Design

Primer Premier 5 software was used to design specific probes between forward and reverse primer targeting sequences, and the formation of dimers, hairpin structures, mismatches, etc. between the probe and the reverse primer should be theoretically avoided as much as possible. The design principles are: (1) The probe size is 46-51 bp, GC content is 20-80%, and Tm is 57-80°C; (2) The maximum hairpin score is 9, and the maximum primer-dimer score is set to 9. The maximum poly-X is set to 5, and other parameters are set to default values; (3) The 5’ end of the probe is labeled with FITC, and the 3’ end was blocked with SpC3, the base at the middle of the probe was replaced with tetrahydrofuran (THF), and there was at least 30 bp of base before the THF site, while at least 15 bp of base followed; (4) The 5’ end of the reverse primer was labeled with biotin.

### RPA-LFS Procedure

The RPA-LFS experiment was performed using the TwistAmp DNA Amplification nfo Kit (TwistDx Inc., Maidenhead, United Kingdom) in 50 μL of the reaction system. The following systems were added sequentially to the lyophilized powder tubes that contained the enzyme components: 29.5 μL rehydration buffer, 2.1 μL forward primer (10 μM), 2.1 μL reverse primer (10 μM), 0.6 μL probe (10 μM), 12.2 μL ddH_2_O and 1 μL template. To ensure that all reaction systems began simultaneously, 2.5 μL of 280 mM magnesium acetate was added to the tube cap, transiently centrifuged, and immediately incubated in a thermostatic heater at 37°C for 30 min. Then, 5 µL of amplification product was used for LFS (Ustar Biotechnologies Ltd., Hangzhou, China) visual detection within 3 min. There are two red lines displayed on the LFS, namely the control line (top) and the test line (bottom). The control line exists in every test to ensure the validity of the LFS, while the test line could only be observed for positive reactions. Each sample has two strips, one for the sample itself and the other for the control.

### Examination of Clinical Specimens

To evaluate the detection compliance of the RPA-LFS method, parallel PCR experiments were carried out to calculate the compliance rates of the results of the two methods. A total of 207 pre-validated CRE clinical strains were collected and bacteria were treated using the heated boiling method. One microliter of the boiled resuspension was taken and used as a template for the RPA-LFS and PCR assays. The compliance rate was calculated as:{(number of positive samples for both methods + number of negative samples for both methods)/total number of samples} × 100%.

## Results

### Design and Screening of Prime Sets for the RPA System

**Figure 1:**
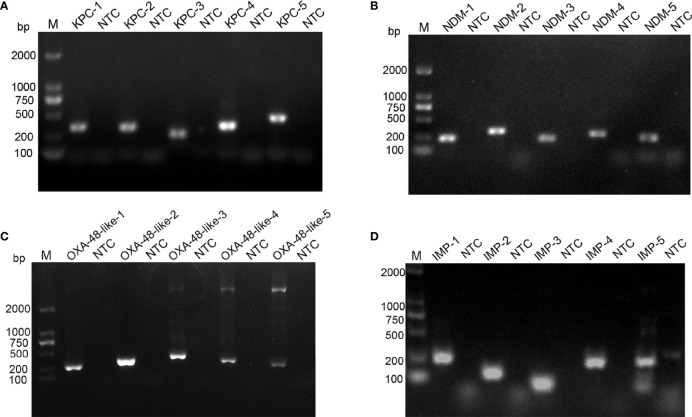
Screening of optimal primer pairs using RPA amplification reactions. Agarose gel images show the amplification results of five primer pairs designed for *bla*_KPC_ **(A)**, *bla*_NDM_ **(B)**, *bla*_OXA-48-like_ **(C)** and *bla*_IMP_ **(D)** genes. The name of each primer pair is above each lane: Lane M is DNA ladder. NTC lanes are template-free controls for the respective primer pairs. Band sizes of the DNA ladders are shown on the left.

The *bla*_KPC_, *bla*_NDM_, *bla*_OXA-48-like_, and *bla*_IMP_ genes were used as target sequences, and five pairs of primer sets were designed for each gene targeting to the highly conserved area (**Supplementary Table 1**). The genome DNA of four standard strains of *K. pneumoniae* were used as templates to verify the amplification of each of the five primer pairs. As shown in **Figure 1**, all designed primers amplified the target bands as expected. Primer pairs with brighter target bands, fewer primer-dimers, without non-specific amplification are of better choice. Therefore, *bla*_KPC_-4, *bla*_NDM_-1, *bla*_OXA-48-like_-2 and *bla*_IMP_-4 were selected for subsequent detection.

### Adding Probes to the RPA-LFS Reaction

**Figure 2:**
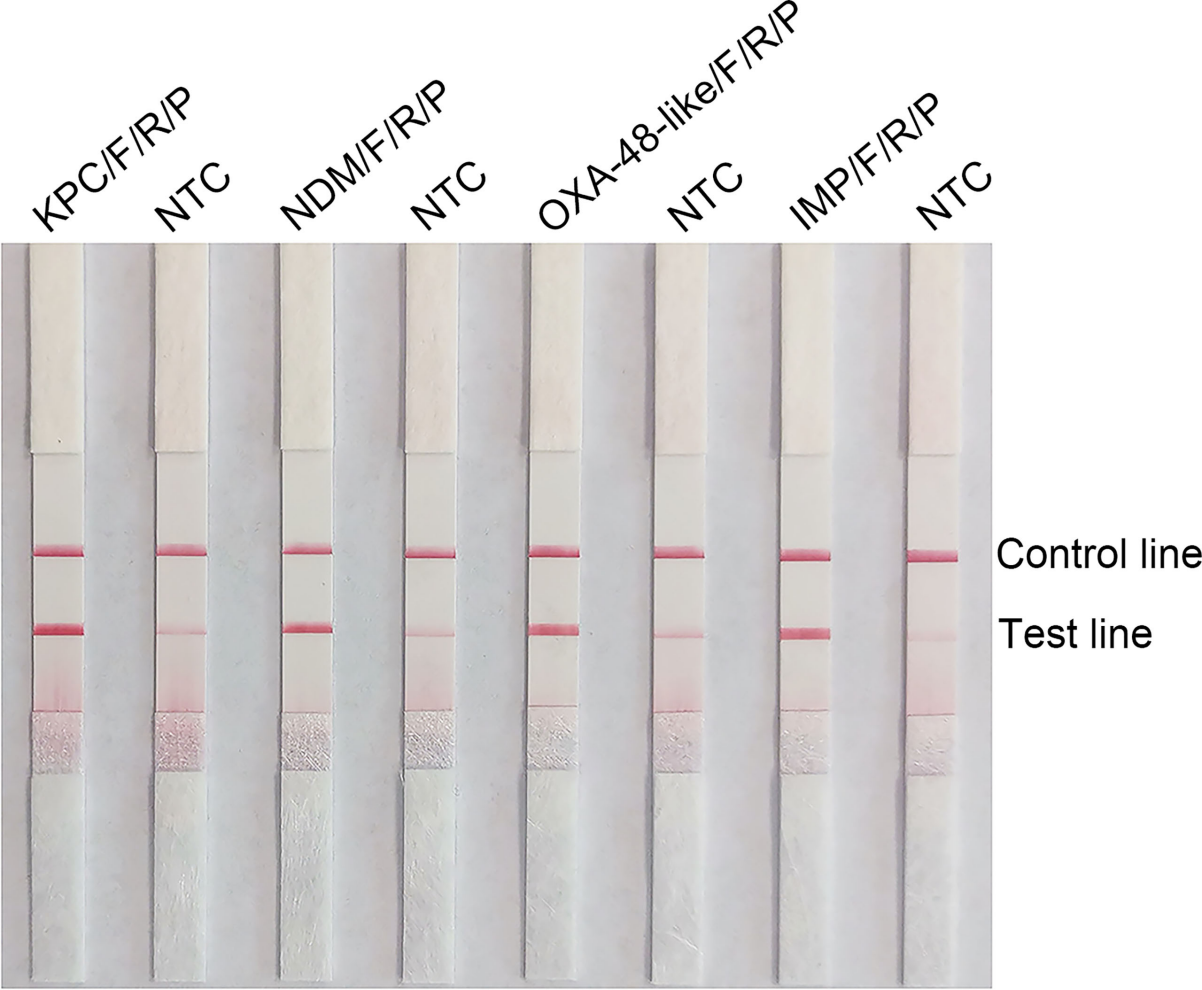
Testing the performance of the primer-probe sets using the RPA-LFS reaction. Results of the LFS detection of the RPA amplification products are shown. The name of each primer-probe set is labeled above the corresponding strip. The NTC strip is the template-free control for the corresponding RPA reaction on the immediate left strip. The positions of the test and control lines are marked to the right of the bars. The image represents the results of three independent experiments.

The use of probes in the RPA reaction increases the amplification specificity and reduces primer-dependent artifacts. Specific probes were designed within the targeting fragments of the four primer pairs that were screened for better performance (**Supplementary Table 1**). The amplification performance and false positives of the primer-probe-needle set were verified. The combination of the four primer-probe pairs provided correct positive signals (two visible red bands both on the test lines and control lines) when tested using RPA-LFS, which indicated that the four primer-probe pairs had good amplification performance. However, they also showed a visible weaken red band on the test line in the no template control, which indicated there are false-positive signals for all these five primer-probe combinations (**Figure 2**).

### Elimination of False-Positive Signals Using Base Mismatches

**Figure 3:**
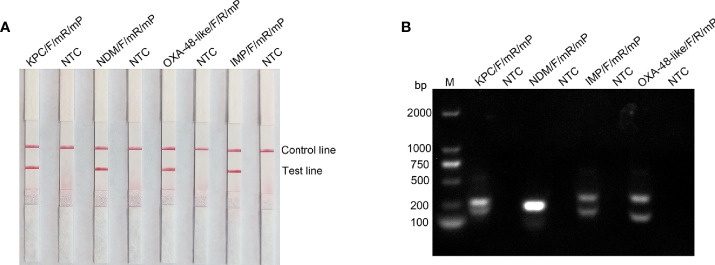
Testing the modified primer-probe set using the RPA-LFS reaction. **(A)** Results of the LFS detection of the RPA amplification products. The name of each primer-probe set is labeled above the corresponding band. **(B)** RPA amplification products were analyzed using agarose gel electrophoresis. The name of each primer-probe set is above each lane: Lane M is DNA ladder. NTC lanes are template-free controls for the respective primer-probe sets. Band sizes of the DNA ladders are shown on the left.

RPA can tolerate some base mismatches between the primer/probe and template, which provides some flexibility in primer/probe design and screening (Daher et al., 2015). Analysis using the Primer Premier 5 software revealed that the probe had multiple consecutively matching bases to the reverse primer, which could result in a false-positive signal. Therefore, base substitutions were introduced to eliminate false-positive signals, and the principles of substitution were (1) Breaks were performed on sites with more than four contiguous matching bases or two or more contiguous matching bases at the 3 ‘ end; (2) no substitution of three bases near the 3’ end; (3) no consecutive two-base substitutions; (4) substitution of preferably no more than three bases, otherwise, the sensitivity of the assay may be affected, and (5) A-G and T-C swaps were used preferentially. The sequences of the modified reverse primer (mR) and probe (mP) are listed in **Table 2**, and the replacement bases are indicated in red. Using this modified primer-probe device, false-positive signals were eliminated without affecting amplification performance (**Figure 3A**). The primer-probe sets were used for all subsequent RPA-LFS reactions. Meanwhile, analysis of the RPA amplification products using 1.5% agarose gel electrophoresis showed that the amplification products of *bla*_KPC_, *bla*_IMP_, and *bla*_OXA-48-like_ primer-probe sets all showed two clear bands, which represented the amplification products of the forward-reverse and probe-reverse primers, respectively, whereas the two amplification products of *bla*_NDM_ were of similar size and, thus, could not be distinguished from each other, so they were shown as a single band (**Figure 3B**).

**Table 2:** Primer-probe sets after base substitution.

Name	Sequence (5’-3’)	Length (bp)	Amplicon size (bp)
KPC-4-F	AACGCCGCCGCCAATTTGTTGCTGAAGGAG	30	269
KPC-4-mR	5’-Biotin-ATGCGGTGGTTGCCGGTCGTGTTTGCCTTT	30	
KPC-mP	5’-FITC-GCGATACTACGTTCCGTCTGGATCGCTGGG [THF]GCTGGAGCTGAACTC-/C3-spacer/-3’	46	196
NDM-1-F	ATGCTGAATAAAAGGAAAACTTGATGGAAT	30	238
NDM-1-mR	5’-Biotin-GCCCCGAAACCCGTCATGTCGAGACAGGAA	30	
NDM-mP	5’-FITC-AATAAAAGGAAAACTTGATGGAATTGCCCA A[THF]ATTATGCACCCGATC-/C3-spacer/-3’	47	232
OXA-2	GTAGACAGTTTCTGGCTCGACGGTGGTATT	30	327
OXA-2-R	5’-Biotin-TTCCTGTTTGAGCACTTCTTTTGTGATGGC	30	
OXA-mP	5’-FITC-TCGAACCTATGATTGGCTGGTGGGTCGGTT [THF]GGTTGAACTTGATGA-/C3-spacer/-3’	46	140
VIM-4-F	TTCATAGTGACAGCACGGGCGGAATAGAGT	30	258
VIM-4-R	5’-Biotin-CGTACGGTTTAATAAAACAACCACCGAATA	30	
VIM-mP	5’-FITC-CAATCCATCCCCACGAATGCGTCTGACTTA [THF]CTAATGAGCTGCTGA-/C3-spacer/-3’	46	217

Modified bases are in red. F represents forward primer, R signifies reverse primer, P means probe and m indicates modified.

### Specificity of the RPA-LFS Assay

**Figure 4:**
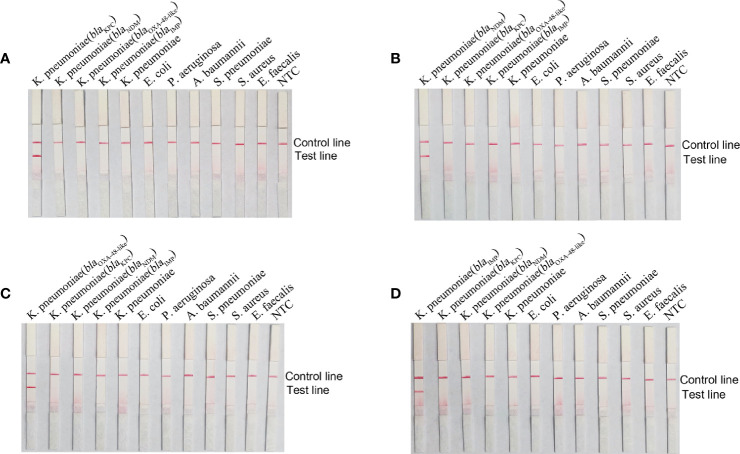
Specificity of the RPA-LFS reaction system. The name of the template added to each reaction is labeled above the corresponding band. Primer-probe combinations of *bla*_KPC_ **(A)**, *bla*_NDM_ **(B)**, *bla*_OXA-48-like_ **(C)** and *bla*_IMP_ **(D)** were added to the reactions. The NTC strip was used as a control reaction without a template.

*K. pneumoniae* genomes carrying *bla*_KPC_, *bla*_NDM_, *bla*_OXA-48-like_, and *bla*_IMP_ genes as well as seven other common pathogenic bacteria were used as templates to detect the specificity of primer-probe combinations. Results are shown in **Figure 4**, all four primer-probe sets could only detect strains containing the corresponding genes, and no bands were present at the test lines when using genomic DNA from other respiratory bacterial pathogens. Overall, these results illustrated that the established RPA-LFS detection system had good specificity towards the four classes of carbapenemases and no cross-reactivity with other pathogenic bacteria.

### Detection Limits of the RPA-LFS Method

**Figure 5:**
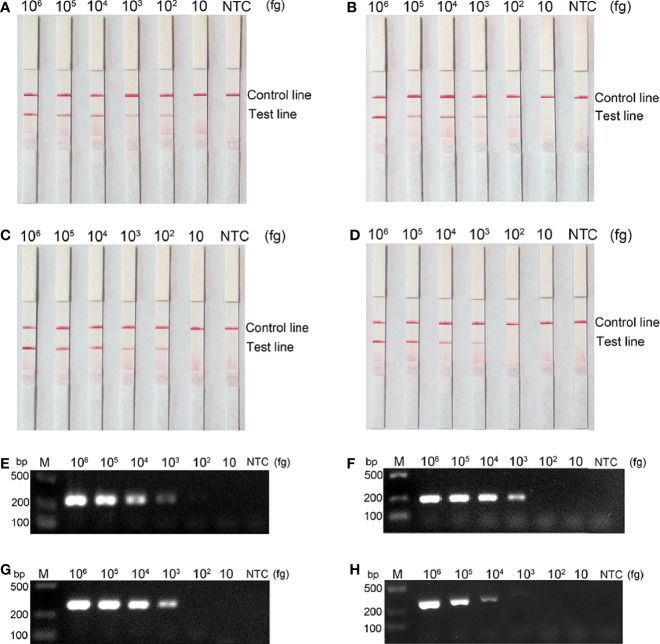
Comparison of the sensitivity between the RPA-LFS reaction and PCR for detection of the four carbapenemase gene families. The amount of template added to each reaction is marked above the corresponding strip. **(A–D)** detection sensitivity of the RPA-LFS assay and **(E–H)** detection sensitivity of the PCR assay, representing *bla*_KPC_, *bla*_NDM_, *bla*_OXA-48-like_ and *bla*_IMP_, respectively. The NTC strip is the control reaction without a template.

To assess the detection limit of RPA-LFS, purified genomes of bacteria carrying the four carbapenemases were subjected to 10-fold serial dilutions, ranging from 10^6^ to 10 fg (50 μL/reaction volume with 1 μL of diluted genome added to each reaction). Results are shown in **Figure 5**. *bla*_KPC_, *bla*_NDM_, and *bla*_OXA-48-like_ all had a minimum detection line of 10^2^ fg/reaction; moreover, *bla*_IMP_ had a 10^3^ fg/reaction. The lowest line for *bla*_KPC_, *bla*_NDM_, and *bla*_OXA-48-like_ was 10^3^/reaction by PCR and 10^4^/reaction for *bla*_IMP_. Our established RPA-LFS reaction system was more sensitive than the PCR reaction.

### Examination of Clinical Strains

The four carbapenemase families of the 207 clinically validated CRE isolates were examined using the RPA-LFS method and conventional PCR. A total of 134 CPE containing the *bla*_KPC_ gene, 69 *bla*_NDM_, 3 *bla*_OXA-48-like_, and 1 *bla*_IMP_ strain (**Table 3**). There was 100% compliance with the two methods of testing. Notably, the RPA-LFS method was simple and rapid and showed a higher proficiency in detecting carbapenemase genotypes in clinical isolates compared with that of conventional PCR and subsequent sequencing.

**Table 3:** Prevalence of carbapenemase genes in 207 strains of *Enterobacterales* using RPA-LFS and PCR.

Method	*bla*_KPC_, n (%)	*bla*_NDM_, n (%)	*bla*_OXA-48-like_, n (%)	*bla*_IMP_, n (%)	Time (min)
RPA-LFS	134 (64.7)	69 (33.4)	3 (1.5)	1 (0.4)	35
PCR	134 (64.7)	69 (33.4)	3 (1.5)	1 (0.4)	100

N represents number.

## Discussion

The emergence of rapid and global spread of carbapenem-resistant *Enterobacterales* (CRE) poses a great threat to human health. Since different types of antimicrobial drugs have different antimicrobial activities *in vitro* against different carbapenemase-producing strains, accurate and rapid detection of carbapenemases produced by CRE is of great value for precise dosing of clinical anti-infective therapy and prevention and control of hospital infection (Bassetti et al., 2016; Sheu et al., 2019; Bush and Bradford, 2019). Several phenotypic methods have been developed for the detection and identification of carbapenemases. Modified carbapenem inactivation method (mCIM) and EDTA carbapenem inactivation method (eCIM): mCIM and eCIM tests are simple to perform, do not require special reagents and are of low cost. The disadvantage is that they both require overnight incubation and is time consuming (Pierce et al., 2017; Lutgring et al., 2018). Combination disk testing (CDT) shows high sensitivity and specificity, but its results take 24 hours and the interpretation of the results is sometimes unclear (Giske et al., 2011). The Carba NP test is simple and rapid (4-6 h) and is suitable for all clinical microbiology laboratories. The disadvantages are low sensitivity to *bla*_OXA-48-live_ and the fact that a certain percentage (3-5%) of the results are uninterpretable (Cunningham et al., 2017). Xpert^®^ Carba-R is rapid and can clarify the carbapenemase genotype. However, special reagents and equipment are required. False negative results will occur if the gene to be tested is different from the target gene (Mancini et al., 2014; Tato et al., 2016). MALDI-TOF has good sensitivity and specificity, but the cost and the requirement for trained personnel limit their use in under-resourced situations (Yu et al., 2018). Enzyme immunochromatographic techniques have the advantage of the simplicity of operation and easy interpretation of results, but the disadvantage is that they are more expensive (Hopkins et al., 2018; Glupczynski et al., 2019).

To cater to the current situation, carbapenemase genes assays should be less expensive and friendly to carry out for end users. The requirements for reduced cost of consumables and technical complexity led to the development of an isothermal RPA assay for the detection of four major carbapenemase families, namely *bla*_KPC_, *bla*_NDM_, *bla*_OXA-48-like,_ and *bla*_IMP_-type. This assay could rapidly amplify the target DNA under low isothermal conditions and tolerate unpurified templates in this complex system, being a promising molecular assay that is user-friendly (no trained staff required), excellent performance (high sensitive and specific), and low cost (approximately $9 per reaction compared to $13.50 for the inmunocromatographic technique and $27 for the Xpert Carba-R technique) (Boutal et al., 2018; Yoon et al., 2021). In addition, the chemical labeling of the RPA reaction allows the amplification product to be read using AuNPs-based LFS in a short time, which does not require highly precise readout equipment.

We initially designed five separate pairs of primers during the development of the primer-probe set for the RPA-LFS assay, however, four were discarded because of the presence of primer-dimers and non-specific amplification. In contrast, the optimal pair of amplification primers was selected to design the probe, although it also produced a false-positive signal on the LFS without the DNA template. Therefore, the introduction of base substitutions on the probe and reverse primer eliminated the false-positive signal. Once the primer-probe device was established, the RPA-LFS method showed good performance in detecting the carbapenemase gene (Daher et al., 2015; Liu et al., 2019). All four primer-probe combinations specifically detected the corresponding genes without cross-reactivity.

Our RPA-LFS system for carbapenemase detection retained the advantageous characteristics of both RPA and LFS technologies. The RPA-LFS method was highly sensitive, requiring only 10^2^ fg of genomic DNA template from a pure culture of carbapenemase bacteria for detection, and was more sensitive than conventional PCR methods, which is consistent with previous studies. Amplification can be performed at 37°C–42°C, and the entire assay can be completed in less than 30 min. When applied to clinical strain testing, samples do not need to be purified, and the DNA is released by simply boiling over heat and used directly in the assay. The detection accuracy was 100%, and the results were consistent with traditional PCR methods. Thus, our RPA-LFS system provides an experimental basis for the rapid detection of carbapenemase genes in clinical strains, which offers justification for the rational clinical use of antibiotics, especially for individualized anti-infective therapy.

There are two limitations of the study. First, although VIM metallo-B-lactamase is also very common worldwide, the detection rate is low in regions surveyed in this study, so the method does not cover VIM metallo-B-lactamase. The use of the test receives some limitations in geographic areas where VIM metallo-B-lactamase is commonly prevalent (Wang et al., 2018; Han et al., 2020). Second, a major limitation of the RPA-LFS method is that amplification of each carbapenemase gene must be performed individually (i.e., 1 reaction/gene), as it is currently difficult to perform multiplex reactions.

## Data Availability Statement

The raw data supporting the conclusions of this article will be made available by the authors, without undue reservation.

## Ethics Statement

The studies involving human participants were reviewed and approved by the Medical Ethics Committee of the Second People’s Hospital of Lianyungang City. The patients/participants provided their written informed consent to participate in the study.

## Author Contributions

FW and WL conceived and designed the experiments. LW, HC, and NL performed the experiments. YW collected the clinical strains. YL analyzed the data. FW wrote the paper. All authors reviewed, revised and approved the final report. All authors contributed to the article and approved the submitted version.

## Funding

This study was supported by grants from the Natural Science Foundation of Jiangsu Province (grant numbers BK20191210) and the “Project 333” training fund of Jiangsu Province (grant numbers BRA2019248).

## Conflict of Interest

The authors declare that the research was conducted in the absence of any commercial or financial relationships that could be construed as a potential conflict of interest.

## Publisher’s Note

All claims expressed in this article are solely those of the authors and do not necessarily represent those of their affiliated organizations, or those of the publisher, the editors and the reviewers. Any product that may be evaluated in this article, or claim that may be made by its manufacturer, is not guaranteed or endorsed by the publisher.
