# Supplementary material for: Rapid Detection of bla KPC, bla NDM, bla OXA-48-like and bla IMP Carbapenemases in Enterobacterales Using Recombinase Polymerase Amplification Combined With Lateral Flow Strip
Source: Front Cell Infect Microbiol. 2021 Dec 2;11:772966. doi: 10.3389/fcimb.2021.772966 (PMC8674914; doi:10.3389/fcimb.2021.772966)
Supplement: Supplementary file 1 [file Table_1.docx]

**SUPPLEMENTARY TABLE 1**

**Supplementary Table 1** Primers and probes tested in this study.

| Name | Sequence(5’-3’) | Amplicon size(bp) |
| --- | --- | --- |
| KPC-1-F | AAATATCTGACAACAGGCATGACGGTGGCG |  |
| KPC-1-R | GGTGGTTGCCGGTCGTGTTTCCCTTTAGCC | 328 |
| KPC-2-F | CGCTTCCCACTGTGCAGCTCATTCAAGGGCTTTC |  |
| KPC-2-R | GGCGGAGTTCAGCTCCAGCTCCCAGCGGTC | 324 |
| KPC-3-F | AACGCCGCCGCCAATTTGTTGCTGAAGGAG |  |
| KPC-3-R | ATGCGGTGGTTGCCGGTCGTGTTTGCCTTT | 269 |
| KPC-4-F | GTGCAATACAGTGATAACGCCGCCGCCAATT |  |
| KPC-4-R | ACTCCGCAGGTTCCGGTTTTGTCTCCGACT | 341 |
| KPC-5-F | CCACTGTGCAGCTCATTCAAGGGCTTTCTT |  |
| KPC-5-R | GGCGGAGTTCAGCTCCAGCTCCCAGCGGTC | 318 |
| NDM-1-F | ATGCTGAATAAAAGGAAAACTTGATGGAAT |  |
| NDM-1-R | GCCCCGAAACCCGTCATGTCGAGACAGGAA | 238 |
| NDM-2-F | ATGCTGAATAAAAGGAAAACTTGATGGAAT |  |
| NDM-2-R | GCCCCGAAACCCGGCATGTCGAGATAGGAA | 238 |
| NDM-3-F | AGCCTGACTTTCGCCGCCAATGGCTGGGTC |  |
| NDM-3-R | CAGCTTGTCGGCCATGCGGGCCGTATGAGT | 233 |
| NDM-4-F | TCGGGGCAGTCGCTTCCAACGGTTTGATCG |  |
| NDM-4-R | CCAGCCATTGGCGGCGAAAGTCAGGCTGTG | 296 |
| NDM-5-F | ATGCTGAATAAAAGGAAAACTTGATGGAAT |  |
| NDM-5-R | AAACCGTTGGAAGCGACTGCCCCGAAACCC | 256 |
| OXA-48-like-1-F | TAAGCAAGGGGACGTTATGCGTGTATTAGC |  |
| OXA-48-like-1-R | GTCCATCCCACTTAAAGACTTGGTGTTCAT | 308 |
| OXA-48-like-2-F | TTAAAATTCCCAATAGCTTGATCGCCCTCG |  |
| OXA-48-like-2-R | AAACTGTCTACATTGCCCGAAATGTCCTCA | 253 |
| OXA-48-like-3-F | GTAGCAAAGGAATGGCAAGAAAACAAAAGT |  |
| OXA-48-like-3-R | GAAATGTCCTCATTACCATAATCGAAAGCA | 389 |
| OXA-48-like-4-F | GTAGACAGTTTCTGGCTCGACGGTGGTATT |  |
| OXA-48-like-4-R | TTCCTGTTTGAGCACTTCTTTTGTGATGGC | 327 |
| OXA-48-like-5-F | GACGTTATGCGTGTATTAGCCTTATCGGCTGTG |  |
| OXA-48-like-5-R | AAATCGAGGGCGATCAAGCTATTGGGAATT | 254 |
| IMP-1-F | CCAGATAACCTAGTAGTTTGGCTGCCTGAA |  |
| IMP-1-R | TTTCGTTTAACCCTTTAACCGCCTGCTCTA | 238 |
| IMP-2-F | TTGTTCCTAAACATGGTTTGGTTGTTCTTG |  |
| IMP-2-R | AGCCACTCTATTCCGCCCGTGCTGTCACTA | 178 |
| IMP-3-F | TTGCTACCGCAGCAGAGCCTTTGCCAGATT |  |
| IMP-3-R | CAAACCATGTTTAGGAACAACGCCCCACCC | 119 |
| IMP-4-F | TTCATAGTGACAGCACGGGCGGAATAGAGT |  |
| IMP-4-R | CGTACGGTTTAATAAAACAACCACCGAATA | 258 |
| IMP-5-F | GCTACCGCAGCAGAGCCTTTGCCAGATTTA |  |
| IMP-5-R | AGCCACTCTATTCCGCCCGTGCTGTCACTA | 275 |
| KPC-3-P | 5’-FITC-GCGATACCACGTTCCGTCTGGACCGCTGGG [THF]GCTGGAGCTGAACTC-/C3-spacer/-3’ | 196 |
| NDM-P | 5’-FITC-AATAAAAGGAAAACTTGATGGAATTGCCCA  A [THF]ATTATGCACCCGGTC-/C3-spacer/-3’ | 232 |
| OXA-48-like-P | 5’-FITC-TCGAACCTAAGATTGGCTGGTGGGTCGGTT  [THF]GGTTGAACTTGATGA-/C3-spacer/-3’ | 140 |
| IMP-P | 5’-FITC-CAATCCATCCCCACGTATGCGTCTGAATTA  [THF]CTAATGAGCTGCTTA-/C3-spacer/-3’ | 217 |
